# Targeted gene suppression by inducing *de novo* DNA methylation in the gene promoter

**DOI:** 10.1186/1756-8935-7-20

**Published:** 2014-08-18

**Authors:** Ai-Niu Ma, Hong Wang, Rui Guo, Yong-Xiang Wang, Wei Li, Jiuwei Cui, Guanjun Wang, Andrew R Hoffman, Ji-Fan Hu

**Affiliations:** 1 King’s Lab, Shanghai Jiao Tong University School of Pharmacy, 800 Dongchuan Road, Shanghai 200240, China; 2 Stem Cell and Cancer Center, First Affiliated Hospital, Jilin University, 519 Dongminzhu Blvd, Changchun 130021, China; 3 Stanford University Medical School, VA Palo Alto Health Care System, 3801 Miranda Avenue, Palo Alto, CA 94304, USA

**Keywords:** Gene suppression, Epigenetics, DNA methylation, Histone code, H3K27 methylation, Gene expression

## Abstract

**Background:**

Targeted gene silencing is an important approach in both drug development and basic research. However, the selection of a potent suppressor has become a significant hurdle to implementing maximal gene inhibition for this approach. We attempted to construct a ‘super suppressor’ by combining the activities of two suppressors that function through distinct epigenetic mechanisms.

**Results:**

Gene targeting vectors were constructed by fusing a GAL4 DNA-binding domain with a epigenetic suppressor, including CpG DNA methylase Sss1, histone H3 lysine 27 methylase vSET domain, and Kruppel-associated suppression box (KRAB). We found that both Sss1 and KRAB suppressors significantly inhibited the expression of luciferase and copGFP reporter genes. However, the histone H3 lysine 27 methylase vSET did not show significant suppression in this system. Constructs containing both Sss1 and KRAB showed better inhibition than either one alone. In addition, we show that KRAB suppressed gene expression by altering the histone code, but not DNA methylation in the gene promoter. Sss1, on the other hand, not only induced *de novo* DNA methylation and recruited Heterochromatin Protein 1 (HP1a), but also increased H3K27 and H3K9 methylation in the promoter.

**Conclusions:**

Epigenetic studies can provide useful data for the selection of suppressors in constructing therapeutic vectors for targeted gene silencing.

## Background

Both basic research and clinical drug development often require the inhibition of the activity of a target gene. Therapeutic antibodies work by blocking the function of proteins, the end products of the gene-mRNA-protein cascade. A variety of antibody drugs have been commercially marketed for the treatment of human diseases [1-3], including anticancer therapy against CD20 (Rituximab, Ofatumubab), CD52 (Alemtuzumab), CD30 (Brentuximab vedotin), CD33 (Gemtuzumab ozogamicin), HER2 (Trastuzumab, Pertuzumab), and EGFR (Cetuximab, Panitumumab, Bevacizumab). RNA interference has been used to inhibit gene function at the RNA level through the dicer-argonaute pathway [4,5]. A major problem with both of these approaches is that neither alters the epigenotype in the promoter, leaving a functioning gene that continues to produce mRNA transcripts. Once antibody or shRNA exposure is terminated, gene expression resumes. Thus, constant exposure to the antibody or shRNA is required for the treatment of disease. Therefore, it would be desirable to design therapeutic drugs that function by permanently blocking the function of the target gene at the DNA level.

It is now possible to target genes by using engineered DNA-binding proteins, such as zinc fingers [6-8], the TALEN (transcription activator-like effector nuclease) proteins [9-12], and the recently-identified CRISPR (clustered regularly interspaced short palindromic repeats) proteins [13,14]. When fused to transcriptional repressors, these DNA-binding proteins can attach to the target gene promoter in order to modulate gene expression. However, transcriptional regulation in eukaryotes is a complex process. Most genes are controlled by the interplay of activating and repressive transcription factors acting at DNA regulatory elements. Thus, in designing targeted transcriptional inhibitors, it is critical to select a potent suppressor domain to coordinate with the guiding protein.

The suppressor domain of the synthetic transcription factor can inhibit the target gene through several distinct epigenetic pathways, including histone modifications (for example lysine residue acetylation and methylation), DNA methylation, and alteration of local chromatin structure. DNA methylation-dependent repression is well established, especially for hypermethylated CpG island promoters that are characterized by a high density of CpG residues [15-18]. However, it is still not known which suppressor will function best for the targeted gene manipulation. To maximize gene suppression, we compared the efficacy of several suppressors for their ability to inhibit the activity of a gene promoter. We were particularly interested in constructing a fusion suppressor that inhibits the gene promoter by harnessing two distinct epigenetic mechanisms.

## Results

### Inhibition of the CMV promoter-luciferase cassette by targeted epigenetic suppressors

**Figure 1:**
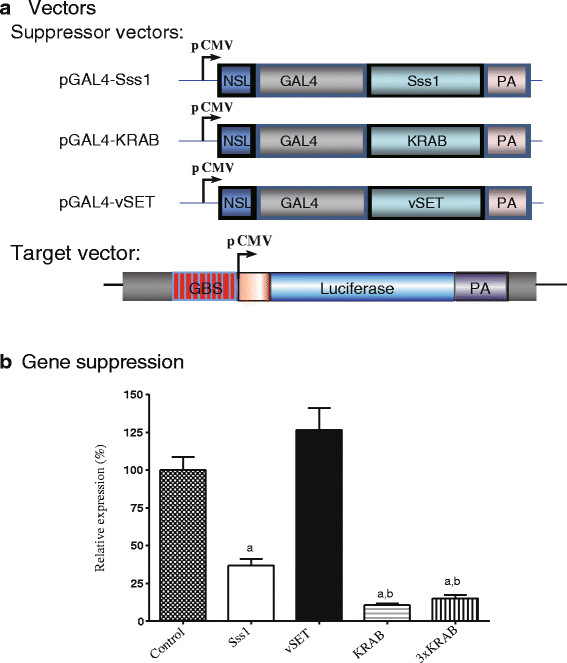
**Targeted suppression of the reporter gene by epigenetic suppressors. a**. Schematic diagram of suppressor and reporter gene vectors. GAL4: the GAL4 DNA binding domain; GBS: GAL4-binding site; KRAB: kruppel-associated box domain; NSL: nuclear localization signal; pCMV: cytomegalovirus (CMV) promoter; PA: SV40 polyadenylation signal; Sss1: methyltransferase gene from Spiroplasma sp. strain MQ1; vSET: the histone H3 lysine 27 methyltransferase SET domain. Synthetic factors use the GAL4 domain to bind to the GBS site in the target gene vector, where the suppressor domain suppresses the activity of the downstream CMV promoter through epigenetic mechanisms. **b**. Relative expression of the reporter gene. 293 T cells were transiently co-transfected with 250 ng suppressor vectors, 250 ng luciferase target vector, and 25 ng thymidine kinase promoter-Renilla luciferase reporter (pRL-TK) control vector. The empty pcDNA3.1 vector was used as the study control. Forty-eight hours post-transfection, cells were harvested for luciferase assay. For comparison, the pcDNA3.1 control vector was adjusted to 100%. Each error bar represents the SEM of three independent experiments. a: *P* <0.05 as compared with the pcDNA3.1 control vector; b: *P* <0.05 as compared with the Sss1 group.

To optimize the potency of different epigenetic suppressors, we constructed a target vector by inserting a GAL4-binding site cluster sequence (GBS) [19] upstream of a cytomegalovirus (CMV) promoter that drives the expression of the reporter luciferase gene (Figure 1A). By measuring luciferase activity, we attempted to determine the best epigenetic suppressor for use in targeted gene silencing.

We constructed a series of suppressor vectors by linking a GAL4-binding domain (GBD) with different epigenetic suppressors, including the CpG DNA methyltransferase Sss1 [20,21], the histone H3 lysine 27 methylase vSET [22,23], and the suppressor domain KRAB [24-26] (Figure 1A). After binding to the GAL4-binding site upstream of the CMV promoter, the epigenetic suppressor domains should inhibit the expression of luciferase through different epigenetic mechanisms.

We transiently co-transfected the GBS-pCMV-luciferase vector and suppressor vectors into 293 T cells. Two days after transfection, CMV promoter expression was determined by measuring luciferase activity. We found that Sss1, a CpG DNA methylase, significantly inhibited the activity of the CMV promoter (Figure 1B). Similarly, KRAB, a Kruppel-associated box (KRAB) domain responsible for the DNA binding-dependent gene silencing activity of hundreds of vertebrate zinc finger proteins, was also very effective in suppressing the expression of the CMV promoter. Interestingly, a three KRAB unit module did not decrease gene expression more than the single KRAB unit construct. However, we did not observe a significant inhibition of the CMV promoter by vSET, a known histone H3K27 methyltransferase domain [22,23], in our reporter system.

### Suppression of the reporter gene promoter by a ‘two-hit’ epigenetic approach

**Figure 2:**
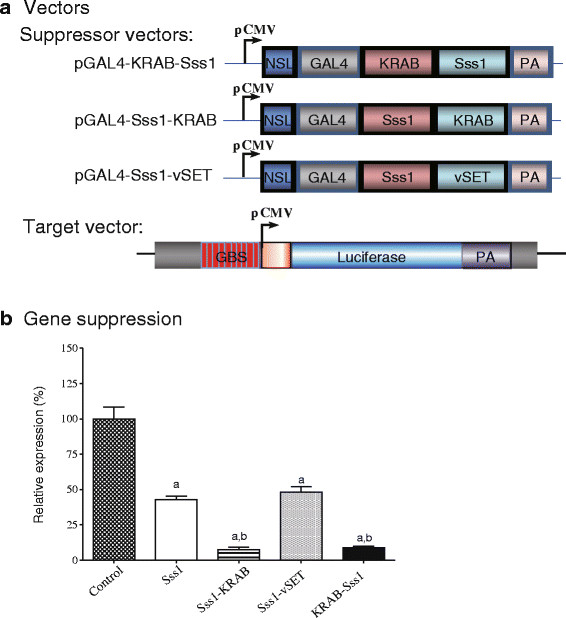
**Suppression of the reporter gene by epigenetic ‘two-hit’ suppressors. a**. Schematic diagram of the two-hit suppressor vectors. Two epigenetic suppressor domains are fused with the GAL4 domain. After binding to the target vector, the synthetic factors suppress the target gene using two distinct epigenetic pathways. **b**. Relative expression of the reporter gene. Forty-eight hours post-transfection, cells were harvested for luciferase assay as described in the Figure 1 legend. Each error bar represents the standard error of mean (SEM) of three independent experiments. a: *P* <0.05 as compared with the pcDNA3.1 control vector; b: P <0.05 as compared with the Sss1 group.

Gene suppressors tested in our system inhibit their target genes using distinct epigenetic mechanisms. We were curious if these epigenetic suppressors can be engineered as a super suppressor that would inhibit target genes at the maximum activity. We then tested the suppressive activity of a vector containing both DNA methylation and H3K27 methylation activities. We constructed three fusion suppressors and tested their potency in 293 T cells (Figure 2A).We first fused CpG methylase Sss1 with H3K27 methyltransferase domain vSET. After co-transfection with the reporter vector, we did not observe an additive or synergistic effect of these two epigenetic suppressor domains (Figure 2B), probably because of the weak activity of vSET in our system (Figure 1B).We also examined the suppressive effect of combining the DNA methylase Sss1 with KRAB. We constructed two fused targeting vectors as Sss1-KRAB and KRAB-Sss1 expression cassettes. Both fusion cassettes showed a significantly higher inhibition rate of target gene expression than did the Sss1 cassette alone (Figure 2B). There were no significant differences in gene silencing when the Sss1 enzyme was inserted in front of KRAB or at the C-terminus of KRAB. We did not observe enhanced inhibition when the vSET suppression domain was linked to CpG methylase Sss1.

### Inhibition of the copGFP reporter by epigenetic suppressors

**Figure 3:**
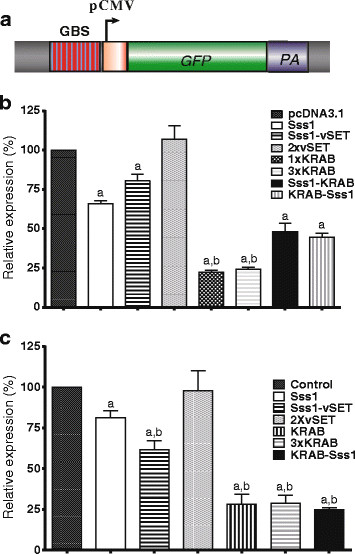
**Epigenetic suppression of the copGFP reporter gene. a**. Schematic diagram of the copGFP reporter gene system. **b**. Inhibition of the transiently-transfected copGFP gene. 293 T cells were transiently co-transfected with copGFP reporter and suppressor vectors. Forty-eight hours post-transfection, copGFP expression was analyzed by luminometer. Each error bar represents the standard error of mean (SEM) of three independent experiments. *Indicates *P* <0.05 versus pcDNA3.1 control vector. **c**. Inhibition of the copGFP gene that has been stably integrated in the genome of the target cell. Expression of the copGFP reporter gene was quantitated by real-time quantitative PCR. Each sample was analyzed in quadruplicate. a: *P* <0.05 as compared with 293 T cells transiently transfected with pcDNA3.1 empty vector; b: *P* <0.05 as compared with the Sss1 group.

In addition to the luciferase reporter, we also examined the inhibition of a second reporter protein copGFP that has a relatively long half-life in host cells (Figure 3A). Using fluorescence microscopy, the suppressors tested showed varying inhibition of the expression of the pCMV-copGFP cassette. Quantitation of copGFP fluorescence revealed an inhibition pattern similar to that seen in the luciferase reporter system (Figure 3B). In general, the KRAB suppressor, whether constructed as a single unit or as three unit modules, showed the best inhibition among the tested domains. The combination Sss1 and KRAB suppressor constructs showed immediate inhibition of the copGFP reporter expression, but the vSET domain cassette did not show significant inhibition in this reporter system.We then used a lentiviral delivery system to insert the pCMV-copGFP cassette into the genome of 293 T cells. The suppressors were transiently transfected into cell clones that stably expressed the copGFP gene. Using this system, we found that the KRAB construct induced the greatest inhibition among the suppressors tested (Figure 3C). The Sss1 insert modestly inhibited the expression of the copGFP inserted genes, while the H3K27 methyltransferase vSET domain did not inhibit the stably-expressed copGFP.

### Epigenetic mechanisms underlying the target gene suppression

**Figure 4:**
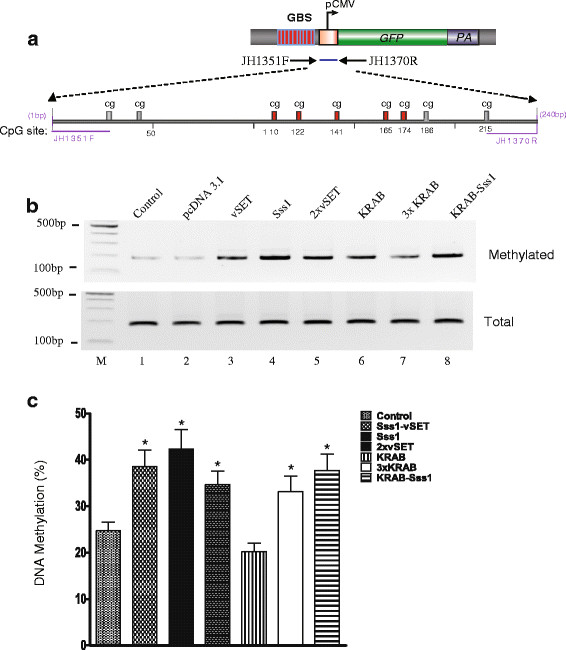
**DNA methylation of the CMV promoter. a**. The schematic diagram of the CMV promoter and the location of cytosine-phosphate-guanine dinucleotide (CpG) islands. After treatment with sodium bisulfite, genomic DNA was amplified with PCR primer JH1351 and JH1370. Red bar: CpG islands that were sequenced. **b**. DNA methylation of the CMV promoter using methylation-specific PCR (MSP). Stable clone cells that have the genomically integrated GBS-pCMV-copGFP were transiently transfected with synthetic suppressor vectors. Genomic DNA was extracted and amplified with primers that specifically recognize the methylated CpGs (top panel). Total genomic DNAs were amplified with primers that recognize both unmethylated and methylated CpGs. **c**. Efficiency of *de novo* DNA methylation by synthetic suppressors. Stable GBS-pCMV-copGFP clone cells were transiently transfected with 1 μg suppressor vectors. Forty-eight hours post-transfection, cells were harvested for bisulfate sequencing. DNA methylation was calculated as the average percentage of methylated CpGs/(methylated CpGs + unmethylated CpGs) from five CpG islands (110, 122, 141, 165, and 174). *P <0.05 as compared with cells transiently transfected with pcDNA3.1 empty vector.

We used several epigenetic approaches to examine how these suppressors inhibit expression of pCMV reporters. We first compared the status of DNA methylation in treated cells using sodium bisulfite sequencing (Figure 4A).In the control cells that received only the reporter vector, there was minimal DNA methylation of CpG dinucleotides near the transcription initiation site (17.6 to 27.5%). In three groups of cells that contain the CpG methylase domain Sss1 insert, there was an increase in CpG methylation of 40 to 60% (Figure 4B, C).

**Figure 5:**
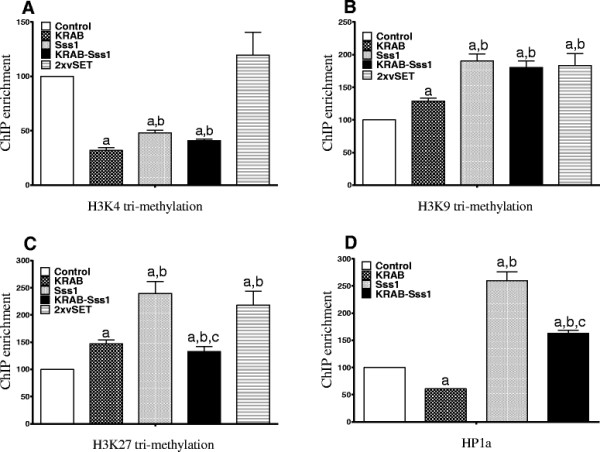
**Promoter histone marks and heterochromatin factor HP1a binding**. Chromatin immunoprecipitation (ChIP) assay was performed with anti-trimethyl H3K4 **(A)**, anti-trimethyl H3K9 **(B)**, anti-dimethyl H3K27 **(C)** and anti-HP1a **(D)** antibodies. Input DNA of each group was amplified by real-time qPCR as a positive control. Stable copGFP clone cells were transiently transfected with 1 μg blank pcDNA3.1 or suppressor vectors. Forty-eight hours post-transfection, cells were harvested for ChIP assay. a: *P* <0.05 versus pcDNA3.1 blank vector; b *P* <0.05 versus KRAB group; and c *P* <0.05 versus Sss1 group.

The 3xKRAB suppressing domain, which uses a different mechanism to inhibit gene activity, also slightly increased CpG DNA methylation as compared with the reporter vector group. When fused with Sss1, CpG DNA methylation significantly increased, presumably reflecting the role of the DNA methylase activity. The induced *de novo* DNA methylation was also confirmed by bisulfite sequencing of a proximal CMV promoter fragment (Additional file 1: Figure S1).We then used a chromatin immunoprecipitation (ChIP) assay to examine promoter histone modifications in the three treatment groups (Sss1, KRAB, and Sss1/KRAB). We focused on histone H3 methylation at lysines 4, 9, and 27 (H3K4, H3K9, and H3K27). H3K4 is associated with an active promoter. We found that treatment with KRAB or Sss1/KRAB significantly reduced H3K4 methylation. Sss1 alone also decreased the level of H3K4 methylation (Figure 5A).Both H3K9 methylation and H3K27 methylation are suppressive markers on gene promoters. Transfection of the KRAB construct enhanced these two suppression signals in the CMV promoter (Figure 5B, C). Surprisingly, Sss1 was the strongest inducer of H3K27 methylation (Figure 5C), in addition to its DNA methylase activity. The vSET domain (2xvSET) increased H3K9 and H3K27 methylation marks in the gene promoter (Figure 5B, C).Heterochromatin Protein 1a (HP1a) functions as an epigenetic ‘gatekeeper’ to inhibit gene activity by binding to H3K9me marks. Using ChIP we found that Sss1, but not KRAB, induced the binding of HP1a to the gene promoter. The KRAB-Sss1 fusion protein recruited HP1a to the promoter at an intermediate level (Figure 5D).

## Discussion

The regulation of gene activity by transcription factors is crucial to the function of all cells. Transcription factors contain a DNA-binding domain that specifically binds to its gene target, and a gene regulatory domain (effector) that either activates or suppresses the promoter activity of the target gene. Accordingly, synthetic transcription factors can be engineered as a powerful tool to regulate the activity of a target gene promoter in mammalian systems, making it a useful approach for either basic research or therapeutic design. This approach could be useful for activating silenced tumor suppressors in antineoplastic therapy, controlling stem-cell differentiation and stimulating tissue regeneration. These synthetic factors can target the promoter of an endogenous gene or be purposefully designed to regulate transgenes. The most common strategies for engineering transcription factors targeted to user-defined sequences have been based on the programmable DNA-binding domains of zinc finger proteins, TELENs, and recently, CRISPR Cas9 system.We compared the action of several synthetic suppressor domains using luciferase and copGFP reporter systems. Through the GAL4 DNA-binding domain, the suppressor domain was tethered to the upstream GBS site of the CMV promoter (Figure 1A). We demonstrate that tethering a KRAB-containing protein and CpG methyltransferase Sss1 triggers epigenetic modifications in the gene promoter and induces suppression of both transiently transfected and stably integrated genes.

Sss1 is a DNA methyltransferase that methylates the C5 position on the base moiety of all cytosine nucleotides contained in unmethylated or hemimethylated double-stranded DNA having the dinucleotide sequence 5’-CpG-3’. Sss1 methylates CpG dinucleotides in a non-specific manner. Once tethered to a target site, it methylates CpG islands in DNA sequences near the region where it binds. Previously, we fused Sss1 to the DNA-binding zinc finger (ZF) domain of chromatin factor CTCF and examined its potential to suppress the promoter of a long noncoding RNA *Kcnq1ot1*. The mouse *Kcnq1* imprinting control region (ICR, or KvDMR_1_) contains two CpG islands: CpG 1 and CpG 2 [27]. The CpG 1 island, located approximately 200 kb downstream of the *Kcnq1* promoter, overlaps with the *Kcnq1ot1* promoter, and contains two critical CTCF binding sites (CTS1 and CTS2). The CpG1 DNA is paternally unmethylated and maternally methylated, thereby allowing the exclusive expression of *Kcnq1ot1* from the paternal chromosome. CTCF binds to the unmethylated paternal allele and may participate in the regulation of the expression of *Kcnq1ot1*[28]. By transfecting the ZF-Sss1 vector into the target cells, we found that the ZF domain guides methylase Sss1 to CTCF sites in the CpG1 island of the paternal allele, and induces *de novo* DNA methylation in the paternal *Kcnq1ot1* promoter. This *de novo* DNA methylation silenced *Kcnq1ot1* and caused the loss of imprinting of its target gene *Kcnq1*[21]. Similarly, in this study we also show that the Sss1 fusion protein can induce *de novo* DNA methylation at the target site (Figure 4), leading to suppression of the reporter gene, whether it is transiently transfected or stably integrated in the host genome (Figure 3).

The Krüppel-associated box (KRAB) constitutes transcriptional repression domains in approximately 400 human zinc finger protein-based transcription factors [29]. The KRAB domain presents one of the strongest repressors in the human genome. KRAB functions through protein-protein interactions via its two amphipathic helices (A box and B box) [26,30-32] and directs the assembly on templates of multiprotein repression complexes containing the primary co-repressor KAP1/KRIP-1/TIF1beta [33]. Upon tethering to specific genomic loci, KAP1 acts as a scaffold for the recruitment of different heterochromatin-inducing factors and complexes, such as Heterochromatin Protein 1 (HP1a), the H3K9me3-histone methyltransferase SETDB1 and the nucleosome remodeling and deacetylase complex NuRD [34,35], accompanied by loss of histone acetylation and an increase of histone 3 lysine 9 trimethylation (H3K9me3) [36]. After tethering to the target promoter, synthetic factors containing KRAB were the most potent inhibitors of the reporter gene in our system (Figures 1, 2 and 3).

Histone H3K9 or H3K27 methylation is normally associated with chromatin compaction [37] and transcriptional silencing [38-40]. The core catalytic domain of these lysine methyltransferases shares a conserved structural fold called the SET domain [41-43]. A conserved SET domain methyltransferase from *Paramecium bursaria* chlorella viruses, termed vSET, uses a ‘walking’ mechanism to suppress host transcription by methylating histone H3 at lysine 27 (H3K27), a mark for eukaryotic gene silencing induction [22]. vSET is the smallest methyltransferase and functions as a dimer [22], in a sharp contrast to the H3K27 methyltransferase EZH2, which is monomeric and relies on polycomb repressive complex 2 (PRC2) partners to achieve optimal activity [44]. When fused to the GAL4 DNA-binding domain, it effectively inhibits the target promoter in 293 T cells [23]. To our surprise, when this vSET domain was introduced into our system, we did not detect any inhibition of the reporter gene promoter. The lack of suppression could be related to differences in vector construction or some other factor in the promoter region.

Each suppressor uses overlapping epigenetic pathways to suppress its downstream target promoter. KRAB, the most potent suppressor in our system, does not affect the status of DNA methylation in the promoter (Figure 4). In contrast, it reduces the concentration of the active histone mark H3K4 methylation and increases the suppressive H3K9 and H3K27 methylation marks (Figure 5). Sss1, although less potent than KRAB, not only significantly induces *de novo* DNA methylation in the gene promoter, but also alters histone marks in the promoter, decreasing H3K4 methylation and enhancing H3K9 and H3K27 methylation. It also simultaneously recruits the heterochromatin protein HP1a. HP1a proteins are ‘gatekeepers’ of epigenetic gene silencing mediated by lysine 9 of histone H3 methylation (H3K9me). Intriguingly, vSET also induces DNA methylation in the promoter (Figure 4), but this does not completely translate into the gene suppression. Based on bisulfite sequencing data (Additional file 1: Figure S1), it appears that the vSET domain induces DNA methylation in a random manner, and it is possible that this random DNA methylation may not be sufficient to block promoter activity. Further studies are needed to address this issue.

Theoretically, the two-hit approach using the Sss1-KRAB fusion construct should yield more compete suppression than the single KRAB construct. However, our data did not demonstrate a significant synergy using the fusion construct. Moreover, it is noteworthy that KRAB alone results in significant amounts of DNA methylation of the gene promoter, which compares well with the levels that are achieved simply by tethering SssI itself (Figure 4, Additional file 1: Figure S1). This observation could explain why creating a fusion with SssI does not increase the suppressive effect.

It should also be emphasized that our reporter gene cassette contains the most potent CMV promoter. Different promoters vary in their content of the CpG islands, particularly those CpGs located in the critical consensus regulatory sequences of the promoter. In addition, the GAL4 DNA-binding site (GBS) is located far upstream of the promoter. All these variables may significantly affect the degree of promoter suppression by the introduced synthetic factors.

## Conclusions

In summary, our data demonstrate that KRAB and Sss1, when tethered to the gene promoter, significantly inhibit the expression of target genes using distinct epigenetic mechanisms. KRAB suppresses gene expression by altering the histone marks in the gene promoter. Sss1, however, inhibits the target gene by multiple pathways, including *de novo* DNA methylation, H3K27 and H3K9 hypermethylation, and the recruitment of Heterochromatin Protein 1 (HP1a). The two-hit constructs containing both Sss1 and KRAB showed slightly better inhibition than either alone. Further studies are needed to examine if this two-hit approach will be useful in constructing therapeutic vectors that target a disease-related gene promoter.

## Methods

### Construction of reporter and effecter vectors

Suppressor domains, including Sss1 (DNA CpG methylase), vSET (histone H3K27 methyltransferase), and KRAB (a Kruppel-associated box domain responsible for the DNA binding-dependent gene silencing activity of vertebrate zinc finger proteins), were amplified and joined with GAL4 DNA-binding domain using PCR overlapping primers (Additional file 2: Table S1). Sss1 was amplified from our CTCF-Sss1 vector [20,21]. The KRAB domain of the human kox-1 gene was amplified from a 293 T cell cDNA sample. The vSET domain was synthesized by PCR using overlapping oligonucleotides synthesized at the Stanford University PAN facility. The GAL4 DNA-binding domain was amplified from pBIND vector (CheckMate Mammalian Two-Hybrid system, Promega, Wisconsin, United States) with primers that carry the linker sequence. The PCR products were gel purified and ligated using PCR to form full length GAL4-suppresor inserts. After digestion with restriction enzymes Xba1 and Apa1, the inserts were ligated into pcDNA3.1 vector (Invitrogen, California, United States).

For the two-hit vectors, the suppressor domains (KRAB, Sss1, and vSET) were ligated with PCR overlapping primers to construct Sss1-vSET, 3xKRAB, Sss1-KRAB, and KRAB-Sss1 suppressor domains. The suppressors were cloned into pcDNA3.1 vector at Xba1/Apa1 sites.

For target gene vector, the GAL4-binding site (GBS) and CMV promoter were amplified by PCR from pACT (Promega, Wisconsin, United States) and pEGFP-N1 (Clontech, California, United States) vectors, respectively, and cloned into a firefly luciferase pGL3 basic vector (Promega, Wisconsin, United States). All vectors were sequenced to validate the sequences. For the GBS-pCMV-copGFP vector, the GAL4 binding site (GBS) was amplified and cloned at the Spe1 site upstream of the CMV promoter in pGreen vector (System Biosciences, California, United States).

### Cell culture and transfection

293 T cells were maintained in Dulbecco’s modified Eagle’s medium (DMEM, Invitrogen, California, United States) supplemented with 10% (v/v) fetal bovine serum (FBS), penicillin (100 U/ml), and streptomycin (100 μg/ml) in a humidified incubator at 37°C and 5% CO_2_. Transient and stable transfections were performed using Lipofectamine™ 2000 (Invitrogen, California, United States). To obtain stable transfection clones, cells were transfected with a GBS-pCMV-copGFP vector and were selected with 5 μg/ml puromycin 48 hours after transfection. Stable clones were selected for examining the suppression of the endogenous copGFP by the constructed suppressor factors.

### Gene activity by luciferase assay

Fresh 293 T cells were seeded into 48-well plates at a density of 1 × 10^5^ cells per well. Suppressor vectors, luciferase reporter vector and pRL-TK control vector were co-transfected into cells using Lipofectamine™ 2000 (Invitrogen, California, United States). Cell lysates were harvested 48 hours after transfection, and dual-luciferase reporter assays were performed using a Turner Biosystems Single Tube Luminometer (Promega, Wisconsin, United States).

### Quantitation of copGFP fluorescence by luminometer

293 T cells were seeded in 12-well plates at a density of 3 × 10^5^ cells per well. Suppressor vectors and copGFP reporter vector were co-transfected into 293 T cells. Forty-eight hours after transfection, lysates were harvested and copGFP expression assays were performed using a Turner Biosystems Single Tube Luminometer (Promega, Wisconsin, United States).

### Real-time qPCR quantitation

Monoclonal 293 T cells carrying GBS-pCMV-copGFP were transiently transfected with suppressor vectors. Cells were harvested 48 hours post-transfection and lysed by TRI Reagent^®^ (Sigma, Missouri, United States). Total RNA was extracted from tissues by TRI-REAGENT (Sigma, Missouri, United States). After DNaseI digestion of total RNA, first-strand cDNA was synthesized by using M-MLV Reverse Transcriptase (Invitrogen, California, United States) as previously described [45,46]. Real-time qPCR was performed using 2xKapa mixed with SYBR (Applied Biosystems, California, United States) on an ABI PRISM 7900 HT Sequence Detection System (Applied Biosystems, California, United States) with coGFP primers (forward, 5’-CCGCCATGGAGATCGAGTG-3’; reverse, 5’-GCCTTTGGTGCTCTTCATCTTG-3’). *β*-*ACTIN* (forward, 5’-CAGGTCATCACCATTGGCAATGAGC-3’; reverse, 5’- CGGATGTCCACGTCACACTTCATGA-3’) was used as an internal control. The assay was repeated in three independent experiments. Each sample was analyzed in quadruplicate. PCR data were normalized to the Ct of *β-ACTIN* as previously described [21,47].

### Methylation specific PCR (MSP)

Genomic DNA was extracted and treated with sodium bisulfite (Sigma, Missouri, United States) as previously described [48-50]. The CMV promoter region was amplified with three primer sets. Universal primers were used to amplify the total DNA (both methylated and unmethylated DNA). Unmethylated DNA-specific and methylated DNA-specific primers (Additional file 2: Table S1) were used to amplify unmethylated and methylated CMV promoter sequences, respectively.

### Quantitation of promoter DNA methylation by bisulfate sequencing

Monoclonal 293 T cells with stable expression of the endogenous copGFP were transfected with the suppressor vectors. After treatments, cells were collected and genomic DNA was extracted. Genomic DNAs were converted by bisulfite sodium using an EZ DNA MethylationGold™ kit (Zymo Research, California, United States) and were purified using a DNA purification kit (Qiagen, California, United States). DNA samples were amplified with PCR primers (JH1351F: 5’-ttttaaagattgtgtatttaaagattg-3’and JH1370R: 5’-aataccaaaacaaactcccattaac-3’) that cover 7 CpG islands. After 2% agarose gel electrophoresis, the predicted bands (240 bp) of the PCR product were recovered using a gel purification kit (Qiagen, California, United States), cloned, and sequenced. DNA methylation was calculated as the average methylation percentage of all CpG sites.

### Promoter histone code by chromatin immunoprecipitation (ChIP)

As described previously [21,51], ChIP assays for histone methylation and HP1a recruitment were performed using an EZ-Magna ChIP™ G chromatin immunoprecipitation Kit (Millipore, California, United States). Briefly, monoclonal 293 T cells for stable expression of copGFP in 10 cm dishes were transiently transfected with 15 μg of various suppressor vectors. Forty eight hours after transfection, cells were cross-linked with 1% formaldehyde (Sigma, Missouri, United States) and harvested for immunoprecipitation. Antibodies used in ChIP assays included anti-H4K4Me3, anti-H3K9Me3, anti-H3K27Me2, and anti-HP1a (Millipore, California, United States). An aliquot of cell lysates was saved to serve as the input DNA control. After the reversal of crosslinking at 62°C for 2 hours and 95°C for 10 minutes, ChIP samples were purified and subjected to real-time qPCR. Individual ChIP assays were repeated three times to confirm the reproducibility of the qPCR. Real-time qPCR was performed using 2xKapa mixed with SYBR (Applied Biosystems, California, United States) on an ABI PRISM 7900 HT Sequence Detection System (Applied Biosystems, California, United States) with pCMV primers (forward, 5’-gcggttttggcagtacatca-3’; reverse, 5’-gggcggagttgttacgacat-3’). Each sample was analyzed in quadruplicate.

### Statistical analysis

Results were expressed as mean ± SEM. Data were analyzed using SPSS software (version 16.0; IBM, New York, United States). Student’s *t*-test or one-way analysis of variance (Bonferroni test) was used to compare statistical differences for variables among treatment groups. Results were considered statistically significant at *P* ≤0.05.

## Abbreviations

ChIP: Chromatin immunoprecipitation; CpG: cytosine-phosphate-guanine dinucleotide; GBS: GAL4-binding site; H3K27: Histone H3 lysine 27; H3K4: Histone H3 lysine 4; H3K9: Histone H3 lysine 9; KRAB: Kruppel-associated suppression box; MSP: methylation specific PCR; pCMV: cytomegalovirus (CMV) promoter; pRL-TK: thymidine kinase promoter-Renilla luciferase reporter; qPCR: Quantitative polymerase chain reaction; SEM: standard error of mean; Sss1: CpG DNA methyltransferase; vSET: the histone H3 lysine 27 methyltransferase SET domain from Paramecium bursaria chlorella virus 1.

## Competing interests

The authors declare that they have no conflicts of interest.

## Authors’ contributions

AM conducted the experiments and carried out data analyses. HW conducted the experiments carried out and data analyses. RG performed DNA methylation and ChIP assays. YW provided the support for the study. WL provided the support for and coordinated the study. JC provided the support for and coordinated the study. GW provided the support for and coordinated the study. ARH provided the support for the study and edited the manuscript. JFH designed the study and wrote the manuscript. All authors read and approved the final manuscript.

## Authors’ information

Andrew R Hoffman and Ji-Fan Hu: these authors are senior authors of this report.

## Supplementary Material

Additional file 1: Figure S1 DNA methylation of the CMV promoter. Stable clone cells were transiently transfected with synthetic suppressor vectors. Genomic DNA was extracted and treated by sodium bisulfite. Total genomic DNAs were amplified with PCR, cloned into pJet vector, and sequenced. Open circles: unmethylated CpGs; solid circles: methylated CpGs. Click here for file

Additional file 2: Table S1 PCR primers used in the study. Click here for file
