# Supplementary figures and images for: Targeted gene suppression by inducing de novo DNA methylation in the gene promoter
Source: Epigenetics Chromatin. 2014 Aug 18;7:20. doi: 10.1186/1756-8935-7-20 (PMC4150861; doi:10.1186/1756-8935-7-20)

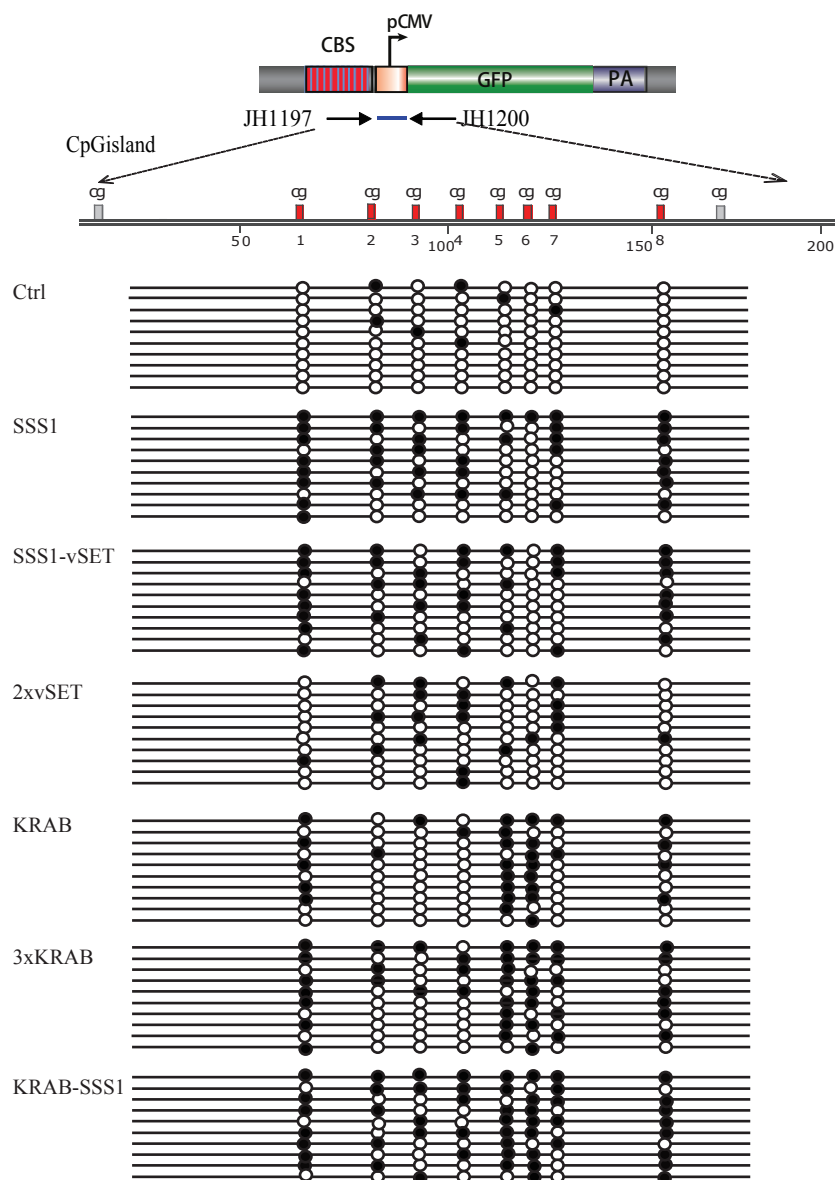

Additional file 1. Induced *de novo* DNA methylation in the CMV promoter

Supplement: Additional file 1: Figure S1 — DNA methylation of the CMV promoter. Stable clone cells were transiently transfected with synthetic suppressor vectors. Genomic DNA was extracted and treated by sodium bisulfite. Total genomic DNAs were amplified with PCR, cloned into pJet vector, and sequenced. Open circles: unmethylated CpGs; solid circles: methylated CpGs. [file 1756-8935-7-20-S1.pdf]
