# Supplementary material for: Targeted gene suppression by inducing de novo DNA methylation in the gene promoter
Source: Epigenetics Chromatin. 2014 Aug 18;7:20. doi: 10.1186/1756-8935-7-20 (PMC4150861; doi:10.1186/1756-8935-7-20)
Supplement: Additional file 2: Table S1 — PCR primers used in the study. [file 1756-8935-7-20-S2.pdf]

SUPPLEMENTAL MATERIALS

Targeted gene suppression by inducing *de no* DNA methylation in the gene promoter

Ainu Ma, Hong Wang, Rui Guo, Yongxiang Wang, Wei Li, Jiuwei Cui, Guanjun Wang, Andrew R. Hoffman, Ji-Fan Hu

Additional file 1. DNA methylation of the CMV promoter. Stable clone cells were transiently transfected with synthetic suppressor vectors. Genomic DNA was extracted and treated by sodium bisulfite. Total genomic DNAs were amplified with PCR, cloned into pJet vector, and sequenced. Open circles: unmethylated CpGs; solid circles: methylated CpGs.

Additional File 2

PCR primers

Gene	Primer	Sequence （5’-3’）	°C
**RT-PCR**			
Actin-F1	J880	CAGGTCATCACCATTGGCAATGAGC	60
Actin-R1	J881	CGGATGTCCACGTCACACTTCATGA	60
GcoGFP-F1	JH1137	CCGCCATGGAGATCGAGTG	60
GcoGFP-R1	JH1138	GCCTTTGGTGCTCTTCATCTTG	60
**Bisulfite DNA PCR**			
pCMV-F1	JH1351	TTTTAAAGATTGTGTATTTAAAGATTG	58
pCMV-R1	JH1370	AATACCAAAACAAACTCCCATTAAC	58
pCMV-F2	JH1197	GGGATTTTTAAGTTTTTATTTTATTGA	60
pCMV-R2	JH1200	TCTAAAATCTTCTATAAAAATCAAAACAA	60
**Methylation-specific PCR (MSP)**			
pCMV meth-F1	JH1351	TTTTAAAGATTGTGTATTTAAAGATTG	62
pCMV meth-R1	JH1536	CCCGTAAATCAAACCGCTATCCACG	62
pCMV unmeth-R1	JH1537	TCCCCATAAATCAAACCACTATCCACA	62
**ChIP PCR**			
pCMV-F1	JH1478	GCGGTTTTGGCAGTACATCA	60
pCMV-R1	JH1479	GGGCGGAGTTGTTACGACAT	60
